# New York Odontological Society

**Published:** 1892-01

**Authors:** 

**Affiliations:** New York Odontological Society


### Reports of Society Meetings.

### NEW YORK ODONTOLOGICAL SOCIETY.

A REGULAR meeting of the New York Odontological Society was held on Tuesday evening, October 20, 1891, at the New York Academy of Medicine, No. 17 West Forty-third Street.

The President, Dr. William H. Dwinelle, in the chair.

### REPORT OF THE RESOLUTION COMMITTEE ON THE DEATH OF DR. JAMES W. WHITE.

The New York Odontological Society learned with painful surprise of the sudden death of Dr. James W. White, of Philadelphia.

Although not a member of this Society, Dr. White has frequently been present with us at our meetings and social gatherings, and his genial face and pleasant voice have left indelible impressions upon the minds of us all. He was a tireless worker; and his literary labors for the advancement of dental science and art have made his name familiar to members of our specialty the world over.

In his native city he was repeatedly honored with positions of trust, which he filled with ability; and his integrity was never questioned. He was recognized and esteemed by his fellow-citizens as a public-spirited man.

The death of such an individual is not only a loss to his family circle, his numerous friends, and to our specialty, but is a public bereavement.

The Odontological Society deplores the departure of this distinguished friend and popular journalist, and its individual members unite in expressions of sympathy for the family and friends of our departed brother.

C. E. FRANCIS.
A. L. NORTHROP.
BENJAMIN LORD.

The report was accepted and ordered spread in full upon the minutes.

Dr. Carl Heitzmann then read the following memoir of Dr. Wedl:

"Many of the members of this Society will recall the venerable figure of the German savant, who, spending a couple of months in the United States in 1882, was tendered a dinner by a number of prominent dentists in this city. His amiable manners, his modesty and kindness, must have struck every one who had the privilege of conversing with him,—the more so as he had remarkable control over the English language. Since Wedl was my first teacher in microscopy, and I have worked with him from 1859 to 1874, the year of my departure from Europe, I beg to furnish here the main data of his life. He fully deserves this honor for what he has done in the science of odontology.

"Wedl was born in Vienna, Austria, on October 14, 1815. In that city he had his collegiate education, receiving the degree of M.D. in 1841. He spent several years in general practice, never finding satisfaction in this calling. After a journey through France and England, in 1844, he determined on the study of histology and pathology, which doctrines, at that time, were in their infancy, nursed by such men as Bichat in France, Addison and Beale in England, Theodor Schwann and Johannes Müller in Germany. Wedl settled in Vienna, and after having published a number of researches, became professor of histology at the University of Vienna in 1853, which chair he held until 1885, when, having reached the seventieth year of his life, according to the law of Austria, he resigned his professorship, ever since living in retirement, almost in seclusion from the world.

"The number of Wedl's publications is large, and on topics widely apart; they were published mainly through the Vienna Academy of Sciences, of which he was a member. We find here a variety of observations on animal and vegetable parasites, among them a description of a fungus growing in dead dentine and bone; a number of valuable contributions to the histology and pathology of the vascular system, blood- and lymph-vessels. His standard work, 'Elements of Pathological Histology,' was published in 1854 in Vienna, and soon afterwards translated into English and edited by the Sydenham Society in London. Two large works of his were issued on the 'Pathology of the Eye,' and a number of pathological observations on the skin and the ear, partly by himself and partly by his pupils. The main work concerning dentistry is his 'Pathology of the Teeth, with Special Reference to Anatomy and Physiology,' published in Leipzig, 1870, and in an enlarged and revised second edition in 1889. The text of this book is German and English; it is accompanied by a number of illustrations, both in the text and in an atlas, the execution of which had been a part of my work during a number of years. The second edition is accessible even at the present time, and ought to be in the hands of every scientific dentist for its intrinsic value.

"Wedl did not meet with many honors, nor with much acknowledgment for what he has done. Lack of ambition and a modesty truly German may account for this fact. Being a bachelor, who had to care only for his nearest kindred, he despised money, and spent a good deal for his yearly travels through Europe, Asia, Africa, and, in 1882, to America. Enjoyments in the line of cuisine or dressing did not exist for him. Nobody who did not know him personally would, upon meeting him accidentally, have taken him for the great savant he really was. His unassuming character is well pictured in his writings. He strictly adhered to the descriptive method, morphology and topography, without the least attempt at speculation or even conclusion. The value of his observations rests, altogether, in the collection of facts, often unbiassed to such a degree that he has been accused by some of lack of system. Nevertheless, his works will remain a rich mine of findings for future reference, in the completion of anatomy and pathology; time and age will never mar the observations of Wedl.

"To me, personally, Wedl has been not only a teacher full of wisdom and good advice, but also a friend with a warm heart and the best of intentions. Last year, while in Vienna, I met him for the last time. He bore his seventy-five years admirably well, being engaged in mathematical and archæological studies, laughing and scorning at the vanities of the world. He told me, smilingly, that he had an aneurism of the upper mesenteric or the splenic artery, which gave him but little distress. It was this disease that caused his death on September 21, 1891, at the age of seventy-six years.

"Blessed be thy memory, dear teacher, and still dearer friend! The scientific value of thy works will be everlasting. Thou wert the truest specimen of the modest, unassuming savant; the very proof that the pursuit of natural philosophy makes the pursuer content and happy."

Voted that the memoir be spread in full upon the minutes of the Society.

The secretary read a communication from Dr. W. C. Barrett, of Buffalo, N. Y., relative to the skull of an ancient Mound-Builder, which he presented to the Society. It was moved that a vote of thanks be tendered to Dr. Barrett; also that the skull, together with the communication accompanying it, be handed to the curator to be labelled and placed in proper position in the osteological collection.

Carried.

*The President.*—It is my melancholy duty to inform you of the death of one of the best of men, Dr. C. A. Kingsbury, of Philadelphia, who passed away a few weeks ago. We shall see him on earth no more. He was a credit to our profession, and he will be greatly missed.

We will now pass to the paper of the evening, entitled "Obscure Pains in and about the Jaws," by Professor J. E. Garretson, of Philadelphia. Gentlemen, I take pleasure in presenting Professor Garretson.

(For Dr. Garretson's paper, see page 8.)

DISCUSSION.

*The President.*—Gentlemen, you have listened to an interesting paper and a very valuable one, in that it contains so many illustrations from Professor Garretson's personal experience. The paper is now open for discussion. Dr. Heitzmann, may we hear from you?

*Dr. Heitzmann.*—Mr. President, if you call on me for discussion, of course you do not expect that I will simply shake hands with Dr. Garretson and say he has read the best essay I have ever listened to. My nature is made of fighting propensities, and I cannot help taking exception to several points in the doctor's paper.

I find there are several things which I want to talk about. Dr. Garretson charged me directly, in speaking of the definition of pain, which he calls altered sensation. Suppose a man has no sensation at all,—so-called sensory paralysis,—what then? Pain is not an altered, but an exalted sensation; it is a plus of sensation, in contradistinction to sensory paralysis, which is a minus or lack of sensation.

*Dr. Garretson.*—Very good; but a man who is in a state of paralysis is not in a state of pain. Again, there is a certain exalted sensation, not entirely unfamiliar to young married men, which does not seem to be esteemed by them particularly painful, judging from their willingness to endure it. As here the very extreme of exalted sensation is referred to, pain cannot be one with exalted sensation. In offering a definition of pain in the term "altered sensation," I define quite as well my lack of knowledge as to what pain is. It is certainly altered sensation inasmuch as it is not natural sensation; the plus or minus of the matter I really know nothing about.

*Dr. Heitzmann.*—Still, sensory paralysis is likewise an altered sensation. If we speak of pain, generally we mean a morbid condition of the sensory nerves. That from the central region, beginning from the strands, down to the finest extremities, any portion of a sensitive nerve, being injured, will cause pain. Hence we are able to understand why that poor fellow Dr. Garretson mentioned, with an exostosis in the foramen ovale, had such terrible pains. It sounds quite reasonable that that man could not localize the pain at all. I know of a case where a lady had such a neuralgia, and localized it in the upper jaw. Tooth after tooth was pulled out, and after all the teeth were gone the neuralgia was as bad as ever.

Dr. Garretson has given us a practical paper. I have not the least reason to militate against what he said from his stand-point, but he gave us also a few morsels of pathology, and there I beg to object to him. He spoke of the inflammation of the lungs. That is called pneumonia. Pneumonitis would be an inflammation of pneumonia; such a word does not exist. He spoke also of Peyer's glands being affected in typhoid fever. These are not glands; they are lymph structures, aggregated lymph-follicles, or lymph-ganglia.

*Dr. Garretson.*—If the gentleman prefers, let "aggregate lymph-follicles" or "lymph-ganglia" be the word. Virchow's definition is, "A lymphatic gland spread out over a surface." I use the term, Peyer's glands, "Peyeri Glandulæ," out of compliment to him who first directed special attention to these bodies. The difference between the doctor and myself is in no sense of pathological import, but wholly of synonyme. Regarding his translation of "pneumonitis," I presume he will discover, perhaps before going to bed to-night, that he has made one of the slips so common to most of us when dealing with the classics. The young members of your Society may have their attention directed, in the connection, to "Thomas's Medical Dictionary," page 554, edition of 1889.

*Dr. Heitzmann.*—Dr. Garretson spoke of a plastic lymph effusion. That is the old view of humoral pathology upon which we were brought up, but which has been considered erroneous for forty years. Virchow, whose seventieth birthday we celebrated a few days ago, overthrew the old theory. It is always an outgrowth of the tissues which makes the inflammation plastic.

Dr. Garretson, from his point of view, brought forth the possibilities existing in and about the teeth, and he beautifully narrated the seven or eight causes which may induce pain about the teeth.

He alluded to caries of dentine, but he did not mention a disease which perhaps is not so well known,—that is, inflammation of the dentine. Since Dr. Bödecker has studied this disease in my laboratory, I can speak about it. Many of the gentlemen present have seen an elephant's tusk which, in the midst of the ivory, had a bullet embedded. We know that the hunter takes as a target the eyes of the elephant, and the bullet sometimes misses its point and buries itself in the tusk, causing an inflammation in the dentine. A foreign body in the dentine works just the same as a splinter would in the skin, or a bullet lodged in the bone. Such a disease, —inflammation of dentine,—eburneitis,—although rare, I have seen occur in human teeth, and it causes excruciating pain. This disease, which Dr. Bödecker described in the *Dental Cosmos* several years ago, I might add to the sources mentioned by the essayist.

If people go to a dentist to have a carious tooth filled, it is because they are afraid of pain and anticipate it. In my youth I was, and still am, quite an energetic fellow, but whenever I suffered from toothache I could not work, and had no relief until the tooth was gone, until I found out that the dentists abroad were too lavish in pulling teeth. The cases which Dr. Garretson mentioned about supernumerary teeth are very interesting.

About two years ago, in this very Society, a tooth was exhibited which had grown from the nasal process into the nasal cavity. Taking into view such possibilities, the great art of the surgeon will be to find out the point where the sensitive nerve is attacked. I know of a case of a Polish Jew who came to Vienna, suffering with what he thought to be neuralgia. Whenever he walked, he had a throbbing in his head. He went to the greatest authorities in Europe, who treated him for neuralgia, and gave him lots of remedies, but no relief. So he came at last to Vienna and was examined very carefully by the best physicians, who could find nothing the matter with him.

The patient was out walking one day, with a friend of his, having his cap on his head as usual, and complaining bitterly about that terrible suffering. His friend turns, and begins to laugh as heartily as possible. The patient said, "Why, what is the matter with you?" The friend answered, "Look; on your cap you wear a tassel, and whenever you walk, that throbs on the front of your head. Remove the tassel, and you will have no more trouble." And so it was.

Neuralgia is an utterly dark field in many cases, and I envy Dr. Garretson for the wonderful faith he has in therapeutics. I

was raised in a sceptic school. I do not believe in remedies as much as Dr. Garretson does, and when he says he gets a cure for erysipelas by using that prescription with tincture of iron in it, it is because the remedy kills the microbe which I am satisfied causes the erysipelas. The air-tight covering of the skin, as in the case of the benzoinated salve, is in many instances sufficient to abate the disease. We imagine that benzoinated salve will cure it; just as often it will cure itself without anything. Faith you must have, of course. The older the practitioner grows the more remedies he has. The essayist reminds me of Dr. Fordyce Barker, one of the presidents of the Academy of Medicine. I was present when he said once that in his family practice there had never been a case of croup or diphtheria, because he makes the mother or the nurse of the child keep a remedy—turpeth mineral, an insoluble salt of mercury—which, if applied, will kill the croup at the very root, and the child cannot have a croup. Of course there was a wild excitement. All ran to the drug store for the remedy he named, the price of which rapidly increased. After a few months the doctors came to him and said it was of no use whatever. Of course the old gentleman was confused to find that it was of no benefit to them. That is the great blessing of faith, for we all know how unreliable medicines are. I was about to say, the older I grow the more sceptical I become.

*Dr. Garretson.*—Accepting myself to be aged in years beyond the distinguished member of your Society who has just spoken, I may hope to be excused in reversing his saying with an assertion that the older I grow the *less* sceptical I am. Principles I long ago discovered to be one with foundation. My dependence is never placed elsewhere. The facts of men must become principles out of reason of standing the tests of analyses before I am willing to consider them. I think I may assume to say that there is not a single fad in the whole thirteen hundred pages of the "System of Oral Surgery," even though I may not hope to have Dr. Heitzmann agree with me regarding primitive dental grooves, tunicæ propriæ, and tunicæ reflexæ. Specifics are not things in themselves, but things of relation with other things. A quarter-dollar buys soup only where soup is to be had. The "Cogito, ergo sum" implies thinker and instrument. Fracture-splints are not, efficiently, fracture-splints save where there are broken bones. Understanding lives with the *process* of *exclusion* and lives never anywhere else. I may, not unwisely, assume that mysteries as to medicines, as indeed to everything else, lie not elsewhere than with ourselves. No truer does it show that cause and effect are relations of a common circle than is exposed the existence of antipodal things: belladonna, to make example, antagonizing opium and opium antagonizing belladonna. Concerning the "plastic lymph exudate," I am simply to reply that I am a humoralist, and refer any who may have interest in my opinion to the seventy-sixth chapter of the fifth edition of my Oral Surgery.

*Dr. Howe.*—I think the Society is to be congratulated on much that Dr. Garretson has said, that will be of great practical value to us. I was sorry that he did not enlarge on some of the points in his address. I hope he will give us the reason for his decision and the way in which he arrived at the conclusion to sacrifice that beautifully-filled tooth he mentioned. He left us rather to infer that he took it out because it was a filled tooth. I am sure there are many here who would like to pump Dr. Garretson—dry if possible—out of his large resources, but it would be a long process, and there is hardly time to-night. One of the things we want to know in our every-day practice is how to find out what is the matter with a patient who comes to us with neuralgia. A patient may have obscure pains about the face, and be referred to the dentist to determine whether the dental factor is concerned in the case. In the absence of ordinary lesions of tooth tissues, the dentist yet knows that there are pulp-stones, exostoses, partial and entire devitalization of pulps, and other obscure causes, which may possibly be involved. What signs or characteristics, if any, are indicative of dental lesions, or how shall we proceed to diagnose the case?

I would like to ask Dr. Garretson whether he coincides in any degree with the view that very many pains are the result of two factors? His reference to malaria and malarial pains in the teeth and to neurasthenic pains in the auriculo-temporal nerve suggests such a thought. In the case of the man whose skull is in the museum, with the spicula of bone projecting from the foramen ovale, the possibilities are that pain did not begin with the very commencement of pressure, but a weakened condition of vitality perhaps was a factor in the irritation. Perhaps I can more plainly suggest what is in my mind by referring to a common tendency to toothache in pregnant women, when an examination of the teeth, or tooth, reveals a lesion altogether inadequate, of itself, to account for the pain. Why should any pain locate in the teeth or be associated with the teeth, from malaria, unless there be a local cause? the latter, alone, often inadequate, but with another and more general factor sufficient, to manifest the irritation in pain.

I would like to ask Dr. Garretson to say a few words on that point before we go, and I would say that if the patient who suffers from neuralgia has been more subject to the pain when going out into the cold wind, or if coming into a warm room, or taking warm or cold food or drink, excites the paroxysms, or aggravates the symptoms, even if the teeth have not ached, I expect almost certainly to find the dental factor concerned in the disturbance. No doubt this indication is well known to most of those present, but I know it to be frequently overlooked by dentists.

*Dr. Garretson.*—In reply to Dr. Howe's first question, I am to say that the "beautifully-filled tooth" was removed by reason of being led to as the probable factor through pursuit of the process of exclusion. Going the round of the circle, the tooth remained as the only thing that I could not demonstrate to myself as not being a cause. If a single possible factor is overlooked in such pursuit of diagnosis through the process ef exclusion, a successful issue out of a case is, of course, not science, but accident.

Answering the second query, I reply that double *dis*es, or factors, are to be accepted as common. Syphilis, as a predisposing cause of jaw pain, must certainly be familiar to one and all; nocturnal exacerbations are reasonably diagnostic. Malaria distinguishes itself through periodicity. The neurasthenic state, as a predisposing, if not, indeed, as the active and sole, cause of neuralgic pains about the face region, is too familiar to me to admit of doubt. The hint lies with the suggestion of Dr. Howe that a localizing factor is more than likely present at the seat of pain,—a hint that is certainly most worthy of being carried in memory to our homes and thought over.

In conclusion, I desire to say to Dr. Howe, and to the members at large of the Society, that I have restricted what has been said by me to narrow limits by reason, first, that I feared to tire the patience of those present; and, second, because I did not want to intrude on time that might be more profitably employed in recital of experiences by others.

Dr. Howe moved a vote of thanks to Dr. Garretson for his interesting and valuable paper.

Carried, unanimously.

Adjourned.

S. E. Davenport, D.D.S., M.D.S.,
*Editor New York Odontological Society.*

